# Prostate SBRT: Comparison the Efficacy and Toxicity of Two Different Dose Fractionation Schedules

**DOI:** 10.3389/fonc.2020.00936

**Published:** 2020-06-25

**Authors:** Donald Blake Fuller, John Naitoh, Reza Shirazi, Tami Crabtree, George Mardirossian

**Affiliations:** ^1^Genesis Healthcare Partners, San Diego, CA, United States; ^2^Consultant, Santa Rosa, CA, United States

**Keywords:** SBRT, CyberKnife, prostate cancer, toxicity studies, dose fractionation

## Abstract

**Background:** CyberKnife SBRT is capable of producing dosimetry comparable to that created by HDR brachytherapy. Our original CyberKnife prostate SBRT schedule of 3,800 cGy/4 fractions (“high dose”) was based upon favorable published prostate HDR brachytherapy experience. Subsequently, our trial was modified to allow a lower dose of 3,400 cGy/5 fractions (“moderate dose”) in selected cases.

**Methods:** Two hundred eighty-nine low and intermediate-risk patients were treated to either high dose (178 pts) or moderate dose (111 pts). The dose selection was individualized; high dose more commonly used in younger, intermediate-risk patients, and moderate dose more commonly used in older, low-risk patients.

**Results:** Median PSA reached 5-year nadir levels of 0.034 ng/mL in the high dose, vs. 0.1 ng/mL in the moderate dose groups, respectively (*p* = 0.044 by year 4), with 62 vs. 44% reaching an ablation PSA nadir (<0.1 ng/mL) by year 5, respectively. Five year biochemical relapse free survival rates measured 98.3% for moderate dose and 94.3% for high dose groups, respectively (*p* = 0.1946). Five-year actuarial grade 2 genitourinary (GU) toxicity rates measured 11.6 vs. 8.7% for high dose vs. moderate dose groups, respectively, with a far lower incidence of grade ≥3 GU and grade ≥2 GI toxicity rates in both groups.

**Conclusions:** Both regimens are efficacious in their respective, selected groups. Both arms have low grade ≥3 GU toxicity and ≥grade 2 GI toxicity. In favor of the original high dose regimen, it has longer follow-up, produces a lower PSA nadir value and is more likely to eventually produce an ablation PSA nadir (<0.1 ng/mL). In favor of the lower dose regimen, it also produces a low PSA nadir, and does so with a slightly lower grade 2 GU toxicity rate. As a lower PSA nadir could be the initial predictor a lower clinical relapse rate far beyond 5 years, even if no difference is apparent within that time frame, a practical strategy could be to more strongly consider the high dose regimen in those with the greatest potential longevity, while for those with a more limited longevity, particularly if they have minimal negative prognostic factors, the moderate dose regimen could be more attractive.

## Introduction

High dose rate (HDR) brachytherapy, using a dose of 3,800 cGy/4 fractions, has shown high efficacy and acceptable toxicity for localized low to intermediate-risk prostate cancer (1). Likewise, “HDR-like” also known as “Virtual HDR” hypofractionated stereotactic body radiotherapy (SBRT) for early-stage prostate cancer has also been reported to have favorable efficacy and toxicity outcomes in mono-institutional and multi-institutional studies (2–4). SBRT delivered in this manner becomes potentially tissue ablative within the high dose zone, a premise that appears to have been confirmed with this specific SBRT dose fractionation regimen, with the median PSA nadir reaching 0.1 ng/mL by 5 years and <0.1 ng/mL by 7 years and with a commensurate high rate of biochemical relapse-free survival at 5 years for low and intermediate-risk patients (3).

The exact prostate cancer α/β ratio has still not been definitively confirmed. If the frequently quoted 1.5 Gy α/β value is correct, it is also likely that lower dose SBRT regimens are effective, whereas is the α/β value is actually higher than that, a larger dose such as that described above will be more effective. Favorable prostate cancer biochemical relapse free rates using lower SBRT doses in the range of 3,500–3,750 cGy/5 fractions have been reported (5–7). A larger pooled analysis of 2,142 men from 10 institutions, treated with 33.5–40 Gy/4–5 fx, demonstrated high 7-year efficacy for low-risk and intermediate-risk disease, with no discernible effect of specific EQD2 on the DFS outcome (8). Until there are a larger volume of long term efficacy data available the optimal prostate SBRT dose fractionation schedule will remain unsettled.

From a dosimetry standpoint, CyberKnife SBRT is capable of producing a dose distribution comparable to that created by prostate HDR brachytherapy treatment, such that our original protocol CyberKnife prostate radiosurgery dose fractionation schedule of 3,800 cGy/4 fractions, was based upon a specific favorable published prostate HDR brachytherapy monotherapy experience (1, 9). Since the inception of our original prostate SBRT study in 2006, excellent biochemical relapse-free survival at other institutions has been reported using lower dose prostate SBRT regimens (5–7). Additionally, experience at our own institution with a lower dose CyberKnife “HDR-like” SBRT regimen of 3,400 cGy/5 fractions for post-radiotherapeutic recurrent prostate cancer has been gained, with favorable PSA response kinetics out to 5 years and acceptable toxicity in spite of their prior pelvic radiotherapy history (10).

Considering this, our original IRB-approved prostate SBRT trial was modified in 2012, to allow our recurrence protocol option (3,400 cGy/5 fractions), hereafter referred to as our “moderate dose” option, to radiotherapy naïve patients without unfavorable intermediate-risk features (our “high dose” option, 3,800 cGy/4 fractions, remains mandatory for all unfavorable intermediate-risk patients). Virtual HDR planning is still applied, regardless of dose prescription (9).

As the number of patients in both dose arms now exceeds 100 (High Dose “*n*” = 178; Moderate Dose “*n*” = 111; Total “*n*” = 289), we report preliminary comparative PSA response, disease free survival, and toxicity results for both dose fractionation regimens.

## Methods

From 2006–2019, 289 patients signed IRB-approved informed consent and were treated on this protocol and fully evaluable, initially on the high dose arm from 2006 to 2012 and thereafter, post-amended protocol, to either high dose or moderate dose, selected on a case by case basis. The dose selection in this trial (3,800 cGy/4 fx vs. 3,400 cGy/5 fx) was not randomized, but rather, left to the discretion of the attending radiation oncologist and patient, after reviewing the potential for greater efficacy in the higher dose regimen vs. the potential for reduced toxicity and improved quality of life (QOL) in the lower dose regimen. Table 1 details the presenting characteristic of patients in each dose group and Table 2 categorizes the specific explanation for dose selection for each patient, from 8 possible reasons.

**Table 1:** Patient characteristics.

		**High dose**	**Moderate dose**	
				***P*-Value**
**Age**	<70	51%	31%	
	≥70	49%	69%	*p* = 0.0015
**Gleason Score**				
	6	38%	50%	
	7	62%	50%	0.0441
**Initial PSA**				
	<10	83%	80%	*p* = 0.6075
	10.0–20	17%	20%	

**Table 2:** Itemized list of reasons for specific dose selection.

**DOSE SELECTION FORM**		
Dose—To be filled out by attending MD (check one):		
High dose: 38 Gy/4 fx________		
Moderate dose: 34 Gy/5 fx________		
REASON for dose selection (check ONE response that captures the reason most accurately)		
**Reason**	**High dose—*****N***	**Moderate dose—*****N***
Pre-protocol amendment (mixed low-risk and intermediate risk cases—high dose is the only option)	83	
Post-protocol amendment: 8 possible reasons		
1. Risk group (high dose is mandated by protocol due to defined high risk factors)	50	
2. Risk group (high dose, though not mandated, is selected due to “non-favorable” risk factors)	35	
3. Risk group (moderate dose is selected due to “favorable” risk factors)		26
4. Age <65 and healthy (high dose is selected due to potential for higher long-term DFS)	10	
5. Age ≥65 or comorbidities (moderate dose selected—more concerned with reduced morbidity)		75
6. Concerned about urinary tract toxicity (moderate dose selected)		7
7. Concerned about GI toxicity (moderate dose selected)		0
8. Concerned about erectile dysfunction (moderate dose selected)		3

The median follow-up is longer in the high dose group (58 vs. 27 months) as this was our original protocol dose, with the moderate dose option added 6 years later. All radiotherapy naïve low-risk (Gleason score 6, PSA <10 ng/mL, T-stage ≤ T2a) and intermediate-risk patients (Gleason score 7 and/or PSA 10.1–20 ng/mL and or ≤ T2b tumor stage) are eligible for the protocol, with no further specific exclusions based on prostate volume or preexisting I-PSS score or any other non-prostate cancer-specific factors. Androgen deprivation therapy is not permitted in this study.

The SBRT volume and intraprostatic dosimetry in our study were made to resemble prostate HDR brachytherapy therapeutic volume as closely as possible, with similar dose escalation dosimetry morphology within the prostate and similar dose limitation objectives to adjacent tissues, including the rectum, bladder, and urethra. Specific dosimetry objectives have been previously described in detail for the high dose regimen (8). The moderate dose arm has the same objectives, scaled to the lower dose. Fiducial-based CyberKnife SBRT technique with continuous real time fiducial tracking was used for all patients. Rectal spacer material (SpaceOAR) became available during the final year of the study and was used in selected patients treated after 2018, representing <3% of total patients analyzed.

**Figure 1:**
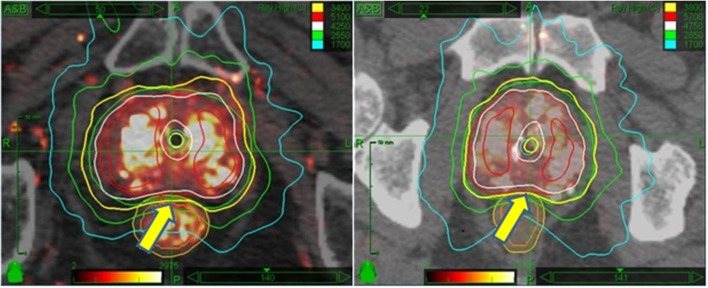
Side by side isodose morphology display: Moderate dose treatment plan **(Left)** vs. high dose plan **(Right)**. In both examples, the 100% isodose line is displayed in yellow, 125% in white, 150% in red, 75% in green, and 50% in turquoise. The major difference is the dose; otherwise both plans are scaled equally to recapitulate HDR brachytherapy isodose morphology. A more subtle difference is the relation of the prescription isodose line to the rectum—allowed to encroach 2 mm into the rectal wall for moderate dose (L panel arrow) vs. 0 mm encroachment for high dose (R panel arrow).

Planning target volume (PTV) margins are based upon the risk and predicted magnitude of extracapsular extension, as reported by Chao et al. (11) To start, a radial margin of 2 mm is added around the prostate in all dimensions to create the planning target volume, subject to additional modification: The margin is focally increased to 5 mm for selected cases as follows: Adjacent to any aspect of the prostate considered to be at elevated risk for subclinical extracapsular extension, including any aspect of the prostate capsule adjacent to any biopsy specimen containing Gleason 7 disease, any aspect of the prostate capsule that contacts abnormal voxels on MRI imaging, and any segment of prostate capsule adjacent to any palpable tumor mass. Proximal seminal vesicle coverage of at least 1.0 cm is added for intermediate risk patients, though full seminal vesicle coverage is not done, as it remains the opinion of the primary author that seminal vesicle >1 cm beyond the prostate is not reliably tracked by intraprostatic fiducial guidance. Where the outer surface of the rectum abuts the posterior surface of the prostate, the PTV margin in that area is reduced to zero for the high dose group, but not for the moderate dose group. This differential posterior margin expansion is based on a concern that the moderate (i.e., – lower) dose group is potentially at higher risk for insufficient posterior periprostatic dose with a zero mm posterior margin expansion, whereas, relatively higher radiobiological potency dose rings extend several mm beyond the PTV in the high dose group. Figure 1 provides a side by side axial display of representative treatment plans for each dose group.

The specific endpoints evaluated and compared in each dose group include PSA response, biochemical relapse free survival rates [Phoenix definition (AKA – “nadir + 2)], grade 2 or higher genitourinary (GU) and gastrointestinal (GI) toxicity rates (CTCAE V 3.0), urinary and sexual quality of life as measured by I-PSS and SHIM score progression, respectively. Follow-up is truncated at 5 years in both groups to allow for a more direct comparison of outcomes at equivalent time points.

## Results

Table 1 reveals a younger age distribution (51 vs. 32% < age 70 for high dose vs. moderate dose groups, respectively, *p* = 0.0015) and higher risk disease distribution in the high dose group (62 vs. 50% Gleason score 7 for high dose vs. moderate dose groups, respectively, *p* = 0.0441), with no significant difference in presenting PSA levels.

### PSA Response Post-SBRT

**Figure 2:**
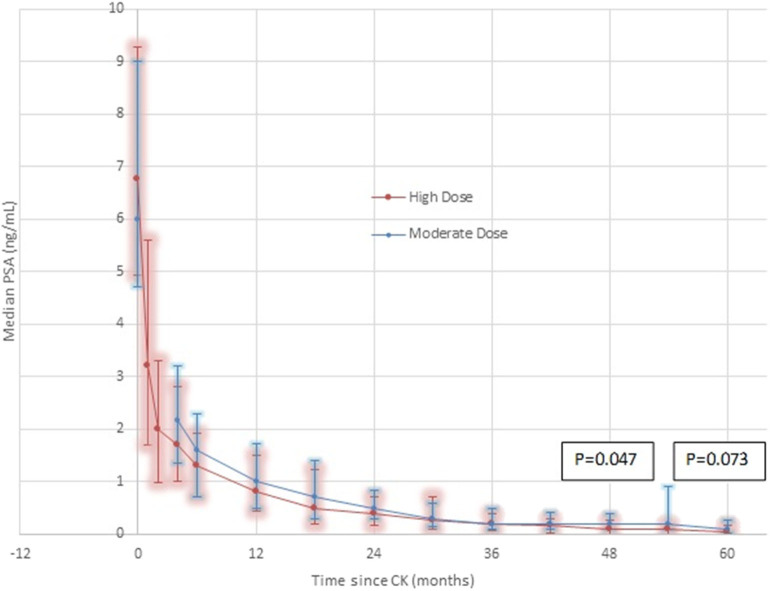
PSA Response by dose group—High Dose vs. Moderate Dose. Although the median 5 year result is very low in both groups (0.1 ng/mL moderate dose, 0.034 ng/mL high dose), there are noteworthy differences. At every comparative time point except 36 months, where they are transiently equal, the median PSA is lower in the high dose group. At every comparative time point except 30 months, the 25th-75th percentile range is lower and with a smaller spread in the high dose group. At 4 years, the difference reaches statistical significance, favoring a lower median PSA nadir in the high dose group, remaining of borderline significance at 5 years. These differences are seen in spite of adverse patient selection in the high dose group, which contains a significantly greater proportion of Gleason score 7 patients and a significantly greater proportion of younger patients, relative to the moderate dose group.

Median Initial PSA levels measured 6.75 ng/mL in the high dose group, and 6.0 ng/mL in the moderate dose group, respectively, with comparable percentage distributions in the <10 ng/mL vs. ≥ 10–20 ng/mL levels in both groups (Table 1). Over time post-CK SBRT, the PSA level decreased in both groups, reaching median levels of 0.8 vs. 1.0 ng/mL at year one, 0.4 vs. 0.5 ng/mL at year 2, and 0.034 vs. 0.1 ng/mL at 5 years in the high dose vs. moderate dose groups, respectively, further detailed in Figure 2. Lower median PSA nadir values were seen in the high dose group at every comparative time point except 3 years, reaching statistical significance at 4 years out (*p* = 0.047) and borderline statistical significance at 5 years out (*p* = 0.073). The percentage of patients reaching an ablation PSA level (<0.1 ng/mL) progressively increases in favor of the high dose group at each annual point after the first year, with the curves diverging thereafter and reaching 62% for high dose patients vs. 44% of moderate dose patients by year 5 (*p* = 0.11).

**Figure 3:**
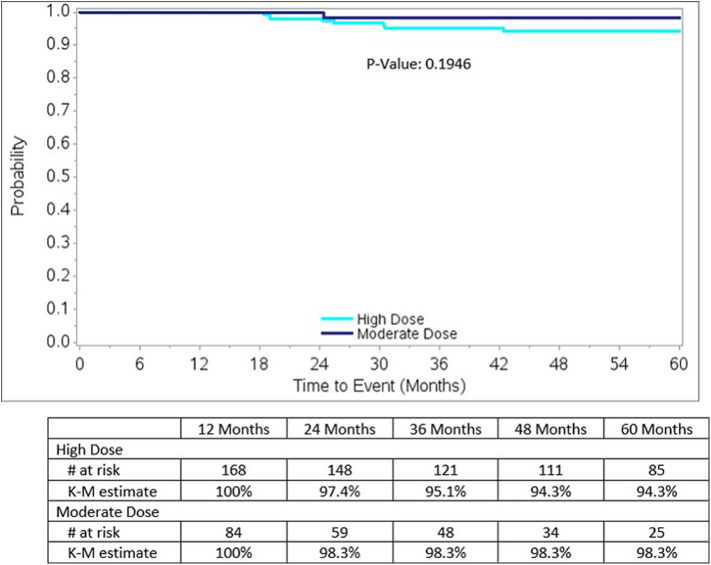
Biochemical Relapse-free survival by dose group for the entire study, nadir + 2 definition: Note there is a significantly higher incidence of Gleason score 7 lesions, 62 vs. 50%, *p* = 0.0441, in the high dose group, relative to the moderate dose group.

Biochemical relapse free survival rates to 5 years for both groups are excellent, as detailed in Figure 3, measuring 98.3% for moderate dose and 94.3% for high dose groups, respectively (*p* = 0.1946). The 5-year rate of local relapse-free survival rate measures 99.2% for the high dose group and 100% for the moderate dose group. The 5-year rate of distant relapse-free survival rate measures 98.3 and 100% for the high dose vs. moderate dose groups, respectively.

**Figure 4:**
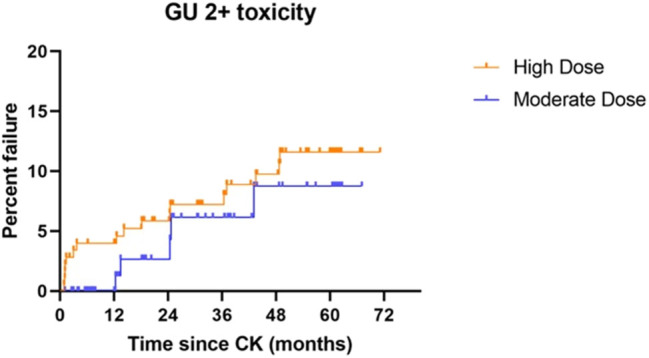
Grade 2 GU toxicity by treatment dose group: Although there is no statistical difference between the groups (*p* = 0.3598), a slightly higher incidence of grade 2 GU toxicity is observed throughout the study period, suggesting the possibility that a difference could emerge with additional patient accrual and follow-up.

Five-year actuarial grade 2 genitourinary (GU) toxicity rates measure 11.6 vs. 8.7% for high dose vs. moderate dose groups, respectively (*p* = 0.3598) while grade 3 GU toxicity rates measure 3.4 vs. 4.5% for high dose vs. moderate dose groups, respectively (*p* = 0.6956). There are no grade 4 or higher GU toxicity events in either dose group. Five-year actuarial grade 2 gastrointestinal (GI) toxicity rates measure 3.0 vs. 4.5% for high dose vs. moderate dose groups, respectively (*p* = 0.7860). There are no grade 3 or higher GI toxicity events in either dose group. Figure 4 illustrates the cumulative grade 2 GU toxicity rates for both dose groups, to 5 years.

Median pre-treatment baseline I-PSS scores measured 7/35 vs. 6/35 in the high dose vs. moderate dose groups, respectively. Post-treatment, both dose groups showed the largest I-PSS increase by 1 month out, reaching a maximum of 12/25 (median increase of 5 I-PSS points) and 14/35 (median increase 8 I-PSS points) in the high dose vs. moderate dose groups, respectively (*p* = NS). Thereafter, median I-PSS scores improved for both dose groups, reaching a low of 2/35 for each group by 3 months out (i.e., – below baseline for both), followed by further modest increase back to baseline minus 1 to baseline plus 3 points at all follow-up intervals thereafter, from 6 months to 5 years out for both groups, with no significant differences between dose groups at any time interval. At 5 years, the median change in I-PSS score relative to baseline is 0 points for the high dose group and minus 1 point for the moderate dose group.

Median pre-treatment baseline SHIM scores measured 12/25 vs. 15/25 in the high dose vs. moderate dose groups, respectively. Post-treatment, both dose groups showed the largest median SHIM score decrease within the first 3 months, decreasing 6 points and 7 points from baseline in the high dose vs. moderate dose groups, respectively (*p* = NS), with continued further gradual decrease in both groups thereafter, with final 5 year median SHIM score decreases of 8 points in the high dose vs. 11 points in the moderate dose groups, respectively (*p* = NS for all comparative time points through year 5).

## Discussion

We see many favorable attributes to HDR brachytherapy, including inherent hypofractionated dose fractionation, which is radiobiologically potent and efficient. Furthermore, there is extremely high conformality and dose customization with this method, which may be harnessed to concentrate the highest dosage in the peripheral zone of the prostate, which normally harbors the greatest density of prostate cancer cells, while simultaneously minimizing dose to the urethra, bladder and rectum. Yet we also note that HDR brachytherapy is an invasive procedure that requires anesthesia and hospitalization to perform, requiring a level of skill that is not reproducible in all centers due to a relative scarcity of HDR experts vs. the need. As such, it is our belief that a non-invasive method with extremely similar dosimetry characteristics would be highly valuable and potentially more reproducible across a large population. We chose our “Virtual HDR” SBRT regimen beginning in 2006 with this core belief as the founding principle.

In the initial creation of our “Virtual HDR” prostate SBRT protocol, we chose to emulate a specific effective published HDR brachytherapy regimen of 3,800 cGy/4 fractions (1). We did this after demonstrating that an “HDR-like” dose distribution could reasonably recapitulated on the CyberKnife SBRT planning computer, escalating the intraprostatic dose to 125–200% of prescribed, while maintaining comparable bladder, rectum, bladder, and urethra Dmax limitation metrics vs. simulated actual HDR brachytherapy (9). This attribute, as well as the published sub-millimeter end to end CyberKnife targeting accuracy, indicating that this complex HDR-like dose distribution is deliverable with high accuracy, caused us to proceed with the treatment of low-risk and selected intermediate-risk prostate cancer patients in this manner under IRB approved clinical trial (clinicaltrials.gov identification number NCT10145148) (9, 12).

Although lower dose, more “uniform dosimetry” prostate SBRT regimens were also initiated at other centers at roughly the same time, such protocols were based on an assumption that prostate cancer is uniquely sensitive to hypofractionation, but with uncertainty regarding that point, and minimal direct experience that those lower dose regimens were effective in actual clinical practice, such that we initially chose to stay with the higher dose regimen. Subsequently, as evidence accrued indicating that lower dose prostate SBRT regimens are also effective, we amended the original protocol in 2012 to allow a lower dose SBRT regimen of 3,400 cGy/5 fractions, which we classified as our “moderate dose” arm, and thereafter allowed patients to be treated under either dose schedule (5, 7, 10).

Both dose arms in this study have a higher equivalent uniform dose (EUD) vs. other commonly reported “homogeneous” prostate SBRT regimens. The EUD with HDR-like intraprostatic dose in the moderate dose arm translates to approximately 125% of the prescribed dose, 4,250 cGy/5 fractions, whereas the EUD of our original high dose regimen is approximately 4,800 cGy/4 fractions. Validating the notion that HDR-like intraprostatic dose escalation increases the potency of treatment, the median PSA nadir in our “moderate dose” (3,400 cGy/5 fraction) arm is 0.1 ng/mL, vs. a median PSA nadir of 0.4 ng/mL reported with a relatively more “uniformly dosed” 3,500–3,750 cGy/5 fraction prostate SBRT regimen, even though its stated dose is nominally higher (6).

### Disease-Free Survival

Eight years after this protocol amendment, we observe that 5 year biochemical disease free survival rates are excellent in dose groups, measuring 94.3% in the high dose group and 98.3% in the moderate dose group, respectively, comparing favorably with other low to intermediate-risk prostate SBRT results (13–15). The minimally higher number in the moderate dose group is likely reflective of case selection rather than any clinically significant difference, as the moderate dose group had a significantly higher prevalence of low Gleason score lesions relative to the high-risk group as detailed in Table 1. Table 2 confirms that the second most common reason for selecting the moderate dose option was the presence of low-risk disease features, whereas the two most common reasons for selecting the high dose option were the presence of higher risk features, thus, confirming a selection bias against the high dose option—done intentionally to maximize the probability of cure for those with higher risk features.

The local-relapse-free survival rate exceeds 99% in both groups, with the more predominant failure pattern being “biochemical only” or distant for the minority of patients that have relapsed. Although this result could also be construed as suggesting that the higher dose arm is unnecessary, considering that the high dose group had definite adverse selection bias and that follow-up is truncated at 5 years, this topic is still not settled, particularly for patients that otherwise have a very long life expectancy.

### PSA Response

Both regimens create a very low PSA nadir by 5 years, 0.1 ng/mL in the moderate dose group and 0.034 ng/mL in the high dose group, respectively, with both results well under any PSA threshold that predicts long term clinical efficacy (16, 17). Of note, there is a statistically significant lower median PSA nadir in the high dose group at 4 years (*p* = 0.047), a borderline significant lower nadir in the high dose group at 5 years (*p* = 0.073), and a widening of the gap in the percentage of patients achieving ablation PSA results (<0.1 ng/mL) between dose groups at each follow-up interval after 1 year, with ablation PSA results achieved in 62 vs. 44% in the high vs. moderate dose groups by 5 years (*p* = 0.11).

Though there is no specific requirement for an “ablation level” PSA nadir post-radiotherapy in the attempted cure of prostate cancer, there are ample data correlating lower PSA nadir levels with improved longer term treatment efficacy (16, 17). Thus, although there is excellent medium term efficacy (≤ 5 years) with essentially all reported prostate SBRT regimens, including both dose arms of the current study, it is possible that shorter term differences in PSA nadir could translate to longer-term differences in late efficacy. In fact this exact result was reported by Zelefsky et al. in the comparison of long term outcomes between prostate IMRT vs. brachytherapy, where the shorter term lower PSA nadir associated with brachytherapy vs. IMRT seen within the first 4 years (0.1 vs. 0.6 ng/mL, respectively) eventually correlated with a lower biochemical disease relapse rate post-brachytherapy, with relapse rate differences not fully observed until 8 years out (18).

A PSA nadir dose response trend has also been reported with SBRT, with one recent study reporting median PSA nadir values of 0.4 vs. 0.1 ng/mL with 35 vs. 40 Gy/5 fx regimens, respectively. Although there was no difference in biochemical relapse rates vs. dose by 5 years, there is a suggestion that such a difference could emerge with larger patient numbers and longer follow-up, as a lower nadir PSA level did predict a lower relapse rate overall (0 vs. 20.5% with nPSA <0.4 ng/mL ≥ 0.4 ng/mL, respectively) (19). An SBRT local control dose response has been suggested more directly by Zelefsky et al. who evaluated sequentially increased SBRT doses from 32.5 to 35.0 to 37.5 to 40.0 Gy in 5 fractions, observing a progressive reduction in the 2-year positive biopsy rate from 47.6 to 7.7% from the lowest to the highest dose regimen. Concurrently, they also reported a reduction in the 5-year biochemical recurrence rate from 15 to 0% progressing from the lowest to the highest dose regimen (14).

Although the difference in median PSA nadir values between dose groups in our current report is of much smaller magnitude vs. that described above, and the absolute PSA nadir in both groups is acceptably low, the fact remains that there is a difference favoring a lower PSA nadir in the high dose group that reaches statistical significance at 4 years, with a progressively higher frequency of ablation PSA nadirs (<0.1 ng/mL) in the high dose group with increased follow-up duration.

Due to the often very protracted natural history of this disease, the PSA response kinetic of a 100% vs. a 99.99% cancer ablation outcome could be similar to identical for well-beyond the endpoint described in this and other “5 year” studies, yet still with eventual dire consequences for “the 99.99% ablation scenario” within a patient's lifetime, particularly for those with the greatest potential longevity. This is a concern that frequently seems to be understated if not omitted entirely in the discussion of prostate cancer treatment efficacy. If therapeutic irradiation is to be considered on a par with radical prostatectomy, it needs to produce a result that will be durable for up to 30 years or more to be presented as a credible alternative to the youngest end of the prostate cancer patient spectrum.

### QOL

This study is not randomized and “concern with potential morbidity” is one of the possible reasons for selection of patients to receive the moderate dose option (Table 2), thus potentially obscuring differences in post-SBRT morbidity issues by preselecting patients with a higher morbidity risk to receive the moderate dose option. Another factor that potentially obscures QOL differences is that the high dose group is younger (Table 1). With these caveats noted, our QOL comparison reveals no significant difference in I-PSS and SHIM score progression between groups out to 5 years.

Regarding urinary QOL, the post-SBRT I-PSS progression between the dose arms is essentially identical, reaching a maximum increase above baseline of comparable magnitude for both groups by 1 month out, with full recovery by 3 months and with a final I-PSS score measuring plus or minus 1 point vs. pre-SBRT baseline by 5 years for both dose groups.

Regarding SHIM score progression, both groups demonstrate similar negative effects, with similarly scaled degradation below baseline over time for both groups; the greatest decrease within the first 3 months and with gradual continued decline thereafter to 5 years. As the moderate dose group is also significantly older at treatment (*p* = 0.0015), this essentially negates any further direct comparison of the sexual domain outcome between dose groups at this time. A more sensitive, full scale EPIC-based QOL comparison could more completely define subtle QOL differences between the dose regimens, but is beyond the scope of this paper. Our study is collecting full EPIC-based data, with the intent to present this as a subsequent stand-alone manuscript.

### Toxicity

The incidence of 5-year cumulative grade 3 GU toxicity and grade 2 or higher GI toxicity is under 5% for both dose groups, with no statistical difference in either domain, a result that is within acceptability bounds vs. other reported radiotherapy toxicity results. In greater detail, for both dose groups in this study, vs. a recent SBRT toxicity incidence report by Zelefsky et al. the incidence of grade 2 GU toxicity is lower (8.6–11.7% in present study vs. 21.1% in the comparison study), grade 3 GU toxicity is minimally higher (3.4–4.5% in present study vs. 2.5% in the comparison study), and grade ≥ 2 GI toxicity is nearly identical (3. 0–4.5% grade 2; 0% grade 3 in present study vs. 3.4% grade 2 and 0.4% grade 3 in the comparison study) (20). The use of pre-rectal spacer material was done very late in the study (final year only). This represents too few patients with too little follow-up duration to make meaningful assessment of any possible further effect of that added step, on the already low rate of observed serious GI toxicity in this population.

Within our own cohort, in the domain of grade 2 GU toxicity, there may be a very slightly higher incidence of cumulative 5-year toxicity in the high dose arm relative to the moderate dose arm (11.6 vs. 8.7%). While not reaching statistical significance, this small difference is observed at essentially every follow-up point from 1 to 5 years out, and thus could become significant with additional patient accrual and follow-up duration. There is no evidence of any difference whatsoever in grade 3 GU toxicity or any grade of GI toxicity between our two different dose cohorts.

## Conclusions

High efficacy and reasonable safety are demonstrated to 5 years with each of the two different prostate SBRT dose fractionation regimens in this protocol, though this conclusion remains more tempered for the moderate dose regimen, due to its smaller sample size, and shorter median follow-up. The 5 year result does suggest that a policy of a stratified SBRT dose assignment based on factors including patient age and risk elements is reasonable. Whereas, the efficacy of high dose “HDR-like” prostate SBRT (3,800 cGy/4 fx) has been previously reported, this manuscript represents the first suggestion of acceptable efficacy with an alternative lower dose “HDR-like” SBRT regimen (3,400 cGy/5 fractions). There are subtle differences worthy of additional consideration.

In favor of the original high dose regimen, it has longer follow-up, produces a PSA nadir value that becomes statistically significantly lower relative to the moderate dose regimen at 4 years, and is the regimen more likely to eventually produce an ablation PSA nadir (<0.1 ng/mL). In favor of the lower dose regimen, it also produces a low 5-year median PSA nadir (0.1 ng/mL), and appears to have a slightly lower grade 2 GU toxicity rate, albeit with no differences in higher grade GU toxicity and no difference in any grade GI toxicity rates, which are low in both arms.

As a lower PSA nadir could be the initial predictor a lower clinical relapse rate far beyond 5 years, even if no relapse rate difference is apparent within that time frame, a practical strategy could be to more strongly consider the high dose regimen in younger patients (e.g., <65 years of age), particularly if they harbor any non-favorable risk factors, and also in those with excellent coexisting health even if they are older, particularly if a family history of significant longevity is also present. On the other hand, for patients with a more limited longevity in the range of 10–15 years, especially if they lack significant negative prognostic factors, the moderate dose regimen could be more attractive. A full 10 year study, with greater patient numbers and longer follow-up in the moderate dose arm would potentially define any efficacy and toxicity differences with greater sensitivity and as such, our protocol continues.

## Data Availability Statement

All datasets generated for this study are included in the article/supplementary material.

## Ethics Statement

The studies involving human participants were reviewed and approved by Scripps Health IRB. The patients/participants provided their written informed consent to participate in this study.

## Author Contributions

DF and JN designed the protocol. DF, JN, RS, TC, and GM analyzed primary data, and are the primary authors of this manuscript.

## Conflict of Interest

DF, JN, RS, and GM were employed by the medical practice known as Genesis Healthcare Partners. DF is a consultant for Accuray. The remaining author declares that the research was conducted in the absence of any commercial or financial relationships that could be construed as a potential conflict of interest.
